# Cooperation and Contagion in Web-Based, Networked Public Goods Experiments

**DOI:** 10.1371/journal.pone.0016836

**Published:** 2011-03-11

**Authors:** Siddharth Suri, Duncan J. Watts

**Affiliations:** Microeconomics and Social Systems, Yahoo! Research, New York, New York, United States of America; Indiana University, United States of America

## Abstract

A longstanding idea in the literature on human cooperation is that cooperation should be reinforced when conditional cooperators are more likely to interact. In the context of social networks, this idea implies that cooperation should fare better in highly clustered networks such as cliques than in networks with low clustering such as random networks. To test this hypothesis, we conducted a series of web-based experiments, in which 24 individuals played a local public goods game arranged on one of five network topologies that varied between disconnected cliques and a random regular graph. In contrast with previous theoretical work, we found that network topology had no significant effect on average contributions. This result implies either that individuals are not conditional cooperators, or else that cooperation does not benefit from positive reinforcement between connected neighbors. We then tested both of these possibilities in two subsequent series of experiments in which artificial seed players were introduced, making either full or zero contributions. First, we found that although players did generally behave like conditional cooperators, they were as likely to decrease their contributions in response to low contributing neighbors as they were to increase their contributions in response to high contributing neighbors. Second, we found that positive effects of cooperation were contagious only to direct neighbors in the network. In total we report on 113 human subjects experiments, highlighting the speed, flexibility, and cost-effectiveness of web-based experiments over those conducted in physical labs.

## Introduction

Why, and under what conditions, presumptively selfish individuals cooperate is a prevailing question in social science that has stimulated an extraordinary range of explanations, many of which have focused on the strategic benefits of cooperation. For example, although displays of altruism may appear to run counter to an individual's self-interest, it is possible to show that if one assumes that individuals possess sufficiently strong other-regarding preferences, then altruism may in fact convey selfish benefits as well [1]. Moreover in a social context, behavior that appears purely altruistic may also accrue individual benefits either because others explicitly reward pro-social behavior [2], [3] or punish selfish behavior [4]–[7]. Finally, individuals may be rewarded indirectly for cooperating, either because a good reputation conveys other transactional benefits [8], or because altruistic behavior can be viewed as a signal of reproductive fitness [9].

In addition to explanations that focus on individual strategies, a longstanding idea is that cooperative behavior might arise as a consequence of the population structure itself [10]. Although initially proposed in the context of evolutionary biology, this idea has particular relevance for social dilemmas among human actors, where the total population is large, but the effects of any one individual's actions fall disproportionately on a relatively small set of neighbors determined either by spatial or social proximity. For example, smog or acid rain causing pollutants disproportionately impact geographically proximate populations; thus one can think of the game as playing out on some approximation of a spatial lattice. Correspondingly, the benefit derived from social networking sites (e.g. Facebook) is highly dependent on the activities and contributions of one's immediate social acquaintances, whose identities in turn depend some complicated mixture of social and spatial distance [11]. Because in either case an individual's neighbors are themselves connected to others, who are in turn connected to others still, and so on, the dynamics of social dilemmas can be thought of as taking place on extended networks [12], [13]. In these settings, outcomes of interest, such as aggregate levels of cooperation, plausibly depend on the structure of the network as well as on the strategies of the individuals in the population [14].

There are two main reasons to suspect that cooperation should depend on network structure. The first reason is that many theoretical models of social dilemmas assume that cooperation is conditional, in the sense that an individual will only cooperate on the condition that its partners are also cooperating. Arguably the clearest example of the principle of conditional cooperation is the celebrated Tit-For-Tat strategy, which has consistently been shown to outperform more exploitative strategies in a range of simulation studies, in large part because it performs well when interacting with other cooperative strategies [15]. In addition, related strategies have also been proposed that generalize the idea of conditional cooperation to multi-player settings [16], [17], usually by specifying some form of threshold requirement—i.e. “I will cooperate if at least X of my neighbors cooperated last round, else I will defect.” Regardless of the specifics of the rule, the implication of these results for networks is that networks characterized by high levels of local clustering [18], meaning that an individual's neighbors are also likely to be neighbors of each other, ought to sustain higher aggregate levels of cooperation than populations in which individuals are randomly mixed [19]. Put another way, local reinforcement would imply that when an individual's neighbors also interact with each other, they are in a better position to reinforce one another's pro-social behavior, and so may be expected to resist “invasion” by defecting strategies better than when each neighbor interacts with a different set of others.

The second reason to suspect that network structure should impact cooperation is that cooperation in networks might be “contagious.” Specifically, if A is a conditional cooperator surrounded mostly by cooperating neighbors, A will cooperate more; but then A's increased cooperation may cause its remaining neighbors to cooperate more as well. These neighbors may in turn cause their neighbors to cooperate more as well, and so on, leading to a cascade of cooperation that sustains itself over multiple steps. In fact, recently it has been claimed that cooperation is characterized by a “three degrees of influence” rule [20], meaning that an individual who increases his or her level of cooperation can positively impact the contribution of an individual who is three steps removed from them in the network. Because the number of individuals who can be reached within three degrees of a cooperating individual will in general depend on the non-local structure of the network [18], the presence of social contagion would imply that network features other than local clustering should also impact aggregate cooperation levels.

Although these heuristic arguments suggest that network structure plausibly impacts cooperation, two other arguments suggest the opposite conclusion. First, even if it is true that unconditional cooperators will benefit from preferential interaction and hence network clustering, conditional cooperation is known to cut both ways, leading as easily to defection as to cooperation [15]. In effect, the assertion that preferential interaction among conditional cooperators will also aid cooperation makes the additional implicit assumption that individuals initially cooperate—an assumption that may or may not hold in practice. Second, the contagion argument implicitly assumes relatively “tight” coupling between neighbors. In coordination games, for example, paired individuals have very clear incentives to choose actions to coordinate with their network neighbors. For example, if A chooses an action that does not coordinate with a one of its neighbors B, then B will have a clear incentive to change its action to accommodate A. If B changes its action, then another of B's neighbors, say C, who is not directly connected to A will nevertheless have an equally clear incentive to coordinate with B as well. In coordination games, therefore, it is easy to see how the influence of one player's action can propagate along chains of intermediaries to affect non-neighbors. And because conditionally cooperative strategies have something of the flavor of coordination games, it is tempting to infer that they lead to the same kind of contagion—indeed it is precisely this intuition that studies like [20] appear to support. However, it is much less clear that individual strategies for resolving social dilemmas do in fact exhibit the same kind of coupling as observed in coordination games, or even should in theory.

In addition to these theoretical arguments, simulation studies of games over networks have also reached mixed conclusions with respect to the impact of network structure on contributions. For example, a number of simulation studies of social dilemmas on spatial lattices [21], [22], and more recently on networks [16], [23], have found that under certain conditions network structure impacts levels. It should be noted, however, that all these results depend on numerous modeling assumptions regarding the behavioral strategies of individual players. Because so many strategies are conceivable, and because the success of conditional cooperation depends on what other strategies are present, it is ultimately inconclusive what can be learned from simulation studies about how real human players will interact in networks.

Finally, experimental evidence concerning the role of network structure is also inconclusive. Although a number of “networked games” experiments have been conducted in recent years using human subjects [24]–[27], they have generally focused on other games, like graph coloring [26], consensus [25], economic exchange [24], and diffusion of social influence [28]. Many of these experiments have found that network structure dramatically impacts collective behavior, consistent with the arguments above. Because all these games differ from one another in subtle but important ways, and because none of them precisely resemble social dilemmas, it remains unclear how these findings can be extended to the question of cooperation on networks. Meanwhile, the extensive experimental literature that explicitly addresses cooperation has largely focused on interactions between pairs [29], or within small, completely connected groups [30]–[32]. To our knowledge, only one experiment has been conducted to test directly for the effects of networks structure, by Cassar [27], who concluded that ‘small-world’ networks (i.e. with high local clustering and short global path lengths) support higher contribution levels in a linear public goods game than randomly connected networks—consistent with the intuition outlined above. For reasons we outline below, however, Cassar's findings were ultimately inconclusive.

As a result of the ambiguous and even conflicting conclusions of previous theoretical, simulation and experimental results, there is a clear need for clarifying experimental evidence. The main substantive contribution of this paper is to investigate the relationship between network structure and cooperation in a series of networked public goods experiments. The experiments we report on were conducted over the World Wide Web using the popular crowdsourcing platform, Amazon Mechanical Turk (http://www.mturk.com). AMT is a web-based labor market originally created to facilitate crowdsourcing [33] of tasks, called human intelligence tasks, or HITs, that are easier for humans than for machines—such as, image labeling, sentiment analysis, or classification of URLs. In addition to its role as a labor market, however, AMT can also be thought of as a convenient pool of subjects willing to participate in laboratory-style behavioral experiments. Mechanical Turk and other web-based experimental platforms are becoming increasingly popular with behavioral science researchers, in part because they allow experiments to be run faster and more cheaply, and in part because they afford access to potentially a much broader cross-section of the population than is typical of university-based lab experiments [34]–[38]. A second contribution of this work, therefore, is to advance the scope of behavioral experiments conducted on AMT to include networked games and more generally, games where all players play simultaneously.

## Results

We conducted a total of 113 experiments on AMT over a period of 6 months. In each of these experiments participants played a widely studied variant of a social dilemma, called a public goods or common pool resource game [39], [40]. Typically such games last for a number rounds, where in each round individuals make voluntary contributions to a common pool. The pool is then augmented in some manner, reflecting the added benefits of the public good. After augmentation the pool is then redistributed to the players, where all players receive an equal share regardless of their contributions. Although many specific variants of this general class of games have been proposed [40], we studied a variant of a standard one in the experimental literature [30], [31], [41] defined by the payoff function 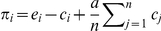 where 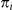 is the payoff to individual 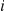, 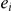 is 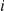
's endowment, 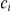 is 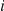
's voluntary contribution, 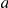 is the amount by which collective contributions are multiplied before being redistributed, and 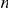 is the group size. Critically, when 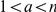, meaning that the marginal per capita return 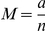 lies in the range 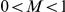 players face a social dilemma in the sense that social welfare is maximized when all individuals contribute the maximum amount, but players have a selfish incentive to free ride on the contributions of others.

### Experimental Design

**Figure 1:**
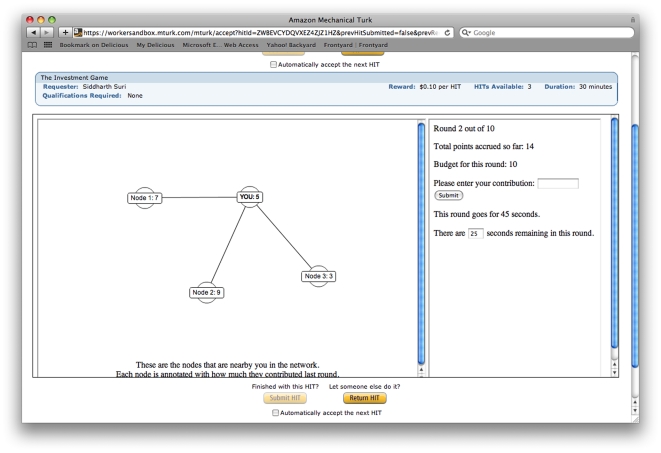
Screen shot of the experiment.

In contrast with standard public goods games, in which participants' contributions are shared among members of the same group, here participants are arranged in a network. To reflect this change, players' payoffs are subject to the modified payoff function 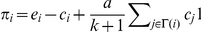, where in place of the summation over the entire group of 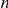 players, payoffs are instead summed over 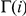, the network neighborhood of 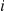 (which we define to include 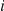 itself), and 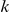 is the vertex degree (all nodes in all networks have the same degree). Therefore, 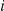
's contributions are, in effect, divided equally among the edges of the graph that are incident on 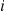, where payoffs are correspondingly summed over 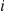
's edges. Aside from this change, our experimental design was kept as similar as possible to previous work, in order to make comparisons possible. Specifically, we ran each experiment for 10 rounds, where the first two rounds each lasted 45 seconds and all subsequent rounds lasted 30 seconds. In each round, each participant received 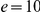 after which they were required to nominate a contribution 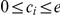 to the common pool. The pool was then augmented and then redistributed to the players, where all players received an equal share regardless of their contributions, as described in the payoff function above. At the end of each round, each player received the following information, which is identical to the information given to the players in [32]: (a) their contribution for that round, (b) the contributions of each of their neighbors for that round, and (c) their own cumulative payoff up to that point. The information visible to players is shown in Figure 1.

**Figure 2:**
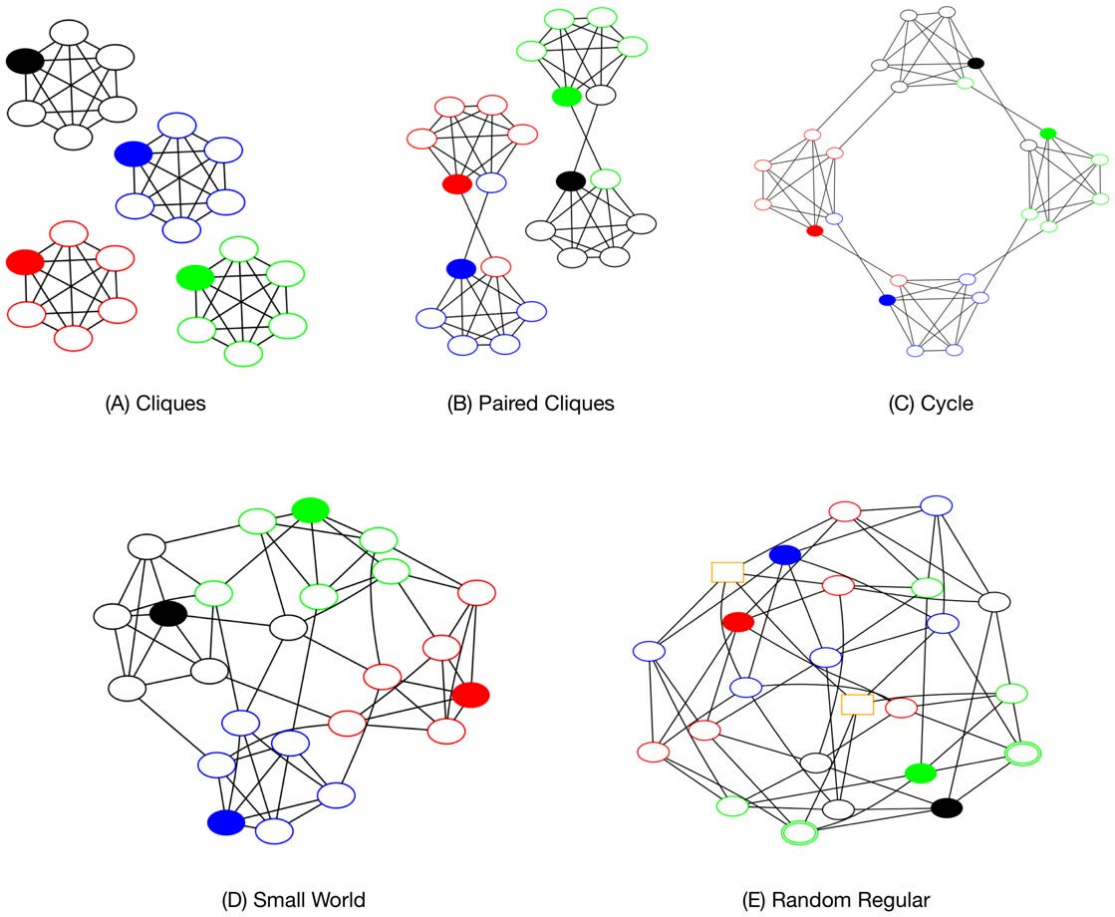
The five networks used in the experiment. (A) four cliques of six players each; (B) two connected components of twelve players constructed by choosing one pair of players in each of two of the cliques in A, and swapping partners; (C) cycle of near-cliques constructed by choosing a pair in each of the four cliques in A and deterministically swapping an edge with a pair from another clique so as form a cycle; (D) “small world” type network formed by swapping four randomly chosen pairs of edges from C; (E) a random regular graph in which all nodes have the same degree 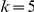. In all cases, the filled in nodes were used as seed nodes in the intervention experiments (see text for details). Each seed node is color-coded, and nodes connected directly to a given seed are outlined with the same color. All nodes in all networks are directly connected to exactly one seed node, except for Random Regular where two nodes are each directly connected to two seed nodes (green double circles) and two nodes are not directly connected to any seed node (orange rectangles).

As shown in Figure 2, we chose networks that spanned a wide range of possible structures between a collection of four disconnected cliques at one extreme, and a regular random graph at the other. All networks comprised 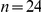 players, each with constant vertex degree 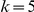; however, they varied with respect to three frequently studied structural parameters, summarized in Table 1: (a) the clustering coefficient 
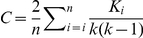
 where 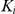 is the number of completed triangles in node 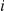
's neighborhood; (b) the average path length 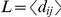 where the average distance between all pairs of nodes is taken over each connected component; and (c) the diameter 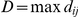, which is the distance between the farthest two nodes. The clustering coefficient of node 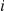 is computed by dividing the number of triangles incident on 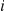 by the number of triangles possible given 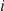
's degree. The clustering coefficient of a network, which is the average clustering coefficient over all nodes, is therefore a local measure of structure that captures the extent to which the neighbors of 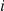 are also neighbors of each other. The average path length and diameter, by contrast, are global network measures that quantify the extent to which effects can propagate along chains of network ties.

**Table 1:** Properties of the five network topologies

	Cliques	Paired Cliques	Cycle	Small World	Random Regular
Clustering Coefficient (C)	1.00	0.80	0.60	0.41	0.09
Average Path Length (L)	1.00	1.81	2.54	2.23	2.01
Diameter (D)	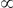	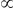	5	4	3
Return on Investment (ROI)	1.04	1.09	1.38	0.80	1.00

In spite of these structural differences, we note that from the perspective of the players, all positions in all networks will seem indistinguishable—players always see themselves interacting with a local network of five others, as in Figure 1. Why then, might we expect the network structure to make any difference? The answer is that although players always play with 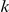 neighbors, the relationship between their neighbors changes as a function of the network. When the network in question is a clique—a set of 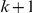 nodes in which every node is connected to every other—our formulation reduces to the standard design in which the group size 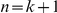. For a general network, however, 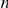 and 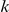 can be specified more or less independently (except that 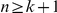), and the connectivity between an individual 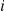
's neighbors can also vary dramatically. In a clique, that is, every neighbor of 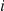 is connected to every other neighbor, whereas in a random graph, 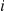
's neighbors will be connected to each other with probability roughly 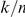 which tends to 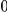 when 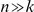. We note that our design differs from previous studies that have compared so-called “partner” vs. “stranger” conditions [32], where in the former condition individuals play with the same partners for multiple rounds, whereas in the latter condition they are randomly rematched on each round. In our design, individuals always play with the same people as in the partner condition. It is the relationship between partners that is different across different network structures. If the “reinforcement” hypothesis, outlined above, is correct, therefore, the actions of an individual's neighbors ought to be dependent on the actions of their neighbors, and hence the experience of the focal individual will depend on the density of interaction between his or her immediate neighbors. Likewise, if the “contagion” hypothesis is correct, the focal individual's experience will depend in addition on the actions of individuals by two or more steps away. Thus our choice of topologies was specifically designed to highlight the importance both of local reinforcement and contagion.

### Recruiting and Retention

The Amazon Mechanical Turk (AMT) community comprises two types of actors: requesters and workers. Requesters can be individuals or corporations, and can list jobs along with a specified compensation. Workers, also known as “turkers,” are paid by requesters to complete individual tasks. When choosing a task to work on, workers are presented with a list of jobs that are subdivided into HITs. Each job contains the title of the job being offered, the reward being offered per HIT, and the number of HITs available for that job. Workers can click on a link to view a brief description of the task, or can request a preview of the HIT. In our case, we posted the experiment as a HIT and recruited workers as subjects to do the experiment. After seeing the preview, workers could choose to accept the HIT, at which point the work was officially assigned to them and they could begin completing the task. HITs range widely in size and nature, requiring from seconds to hours to complete, and compensation varies accordingly, but is typically on the order of $0.01–$0.10 per HIT. Currently, several hundred requests may be available on any given day, representing tens of thousands of HITs (i.e. a single request may comprise hundreds or even thousands of individual HITs). AMT also provides a convenient API for transferring payments from requesters to workers.

Although AMT and other web-based experimental platforms are becoming increasingly popular with behavioral science researchers, the bulk of previous work has relied on experimental designs that are asynchronous, in the sense that they do not require a large group of subjects to participate at the same time. In [42], for example, participants arrived sequentially, and only saw information about the behavior of previous participants, while in [43], at most pairs of participants were required to be present simultaneously. In our experiment, however we required all players to participate simultaneously—a problem that is solved in physical labs by announcing official start times and supervising experiments with trained proctors. To resolve this problem, we instituted a number of web-specific experimental procedures, as described next and in more detail in [38].

#### The Waiting Room

Because it was impossible to assure that participants arrived at precisely the same time, and also because different participants required more or less time to read the instructions and pass the quiz (see below), we created a virtual “waiting room,” similar to [44]. Once they had accepted the HIT and passed the quiz, participants saw a screen informing them that the experiment had not yet filled, along with how many remaining players were required. Once all positions had been filled, participants in the waiting room were informed that the game was about to commence.

#### The Panel

In a series of preliminary experiments, we learned that simply posting the HIT on AMT was insufficient to fill networks of size 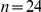 in a reasonable time, resulting in participants abandoning the waiting room and the HIT being terminated. To alleviate this problem, we ran a series of experiments with 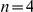, for which waiting times were reasonable, and then at end of each experiment, allowed participants to opt-in to being notified of future runs of our experiment. In this manner, we created a standing panel of 152 players who had played previously and who understood the instructions (i.e. they qualified as experienced players, consistent with previous work [31]). All 113 experiments reported here were conducted using this panel, the self-reported demographic composition of which is reported in Table 2. The evening before any experiments were to be held, we sent messages to the panel (via the AMT API), informing them what time the experiments would be, typically at 11am, 1pm, 3pm and 5pm EST, although other times of day were used in a few instances. We also posted the time of the next days experiments on turkernation.com, a bulletin board site for turkers. At the announced times, participants would log in to AMT, where the first 24 players to read the following instructions and pass the quiz at the end of it were allowed to enter the experiment.

**Table 2:** Self-reported demographic information of panel members.

Gender	Male	61.8
	Female	35.5
	Did Not Answer	2.7
Average Age		32
Highest degree or level of school completed	High School	21.1
	Associates	9.2
	Bachelors	42.1
	Masters	18.4
	Doctorate	3.9
	Professional	3.9
	Did Not Answer	1.4
Race	Asian	26.3
	Black or African American	1.3
	American Indian or Alaskan Native	0.7
	White	69.7
	Did Not Answer	2.0
Marital Status	Divorced	4.6
	Now Married	42.1
	Never Married	49.3
	Separated	2.0
	Did Not Answer	2.0
Total Annual Household Income	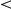 10k	13.8
	10k–20k	13.2
	20k–30k	9.9
	30k–40k	12.5
	40k–50k	15.1
	50k–60k	7.2
	60k–70k	5.3
	70k–80k	4.6
	80k–90k	2.0
	90k–100k	2.6
	100k–150k	6.6
	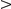 150k	5.9
	Did Not Answer	1.4

#### Handling Dropouts

In spite of these precautions, individual participants would occasionally fail to enter a contribution on one or more turns, or leave the game entirely. In rare instances, a participant who had accepted the HIT and passed the quiz did not participate at all in the game. To handle these circumstances, we adopted the following rules: 1) If a player had entered at least one contribution, and if they subsequently failed to enter a contribution, the system would automatically enter the same contribution as their previous round. 2) If a player did not enter an initial contribution, the system would random choose a contribution of either 0 or 10 for that player (roughly 70% of the contributions during round 1 where either 0 or 10). To avoid biasing our results, we only used data from a given realization if at least 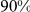 of the contributions in the entire experiment were actually made by human players.

### Calibrating the AMT population

**Figure 3:**
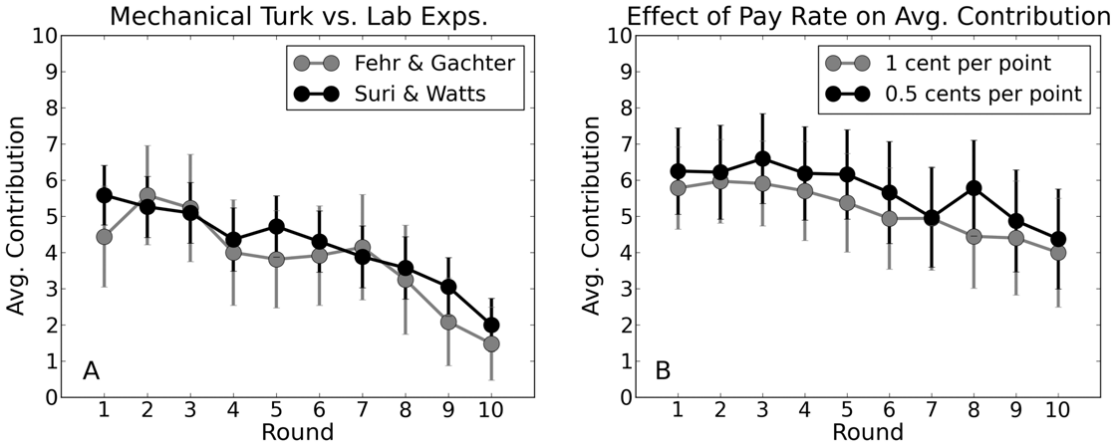
Calibrating the AMT platform. (A) Comparison of contributions for identical linear public goods games conducted on Amazon Mechanical Turk and in a physical lab [32]. (B) Contributions for different compensation levels. In both panels error bars indicate 95% confidence intervals.

Before proceeding with our main results, we first address two legitimate sources of skepticism regarding web-based experiments. First, subjects playing at home or at work may behave systematically differently from those playing in a physical lab; thus the results obtained in a web environment may not be comparable to those obtained in lab-based studies. To address this issue, we conducted a series of 24 preliminary experiments that were designed to replicate the conditions of a previous lab-based study [32]. Specifically, we arranged the players in completely connected groups (cliques) of 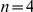 (equivalent to 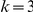) and set 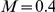. One difference between our design and [32] was that per-round endowments in our experiment were 10 points, instead of 20. Normalizing for these different endowments, however, Figure 3A shows striking agreement between the two sets of results, where we note that qualitatively similar average contribution levels have also been found in other experimental studies [31]. A second issue is that the compensation rates in AMT are substantially lower than in traditional lab experiments; thus one might suspect that subjects are correspondingly less motivated to play seriously. Previous studies such as [45] have shown that for these types of economic experiments, paying a low or high rate does not have a large impact on results as long as the payoff amount has a nonzero dependency on performance. Nevertheless, we conducted an additional series of 16 experiments which alternated the compensation between $0.01 per point and $0.005 per point (participants were also paid a fixed up-front fee of $0.50 for accepting the task and passing the quiz). As Figure 3B shows, contribution levels for both compensation levels were similar, which is also consistent with prior work [45]. We therefore conclude that neither compensation rates nor context significantly affected the behavior of subjects in our games, relative to previous studies. Thus reassured, we now proceed to discuss our main results, which concern behavior on networks.

### Testing for Effects of Network Structure

**Figure 4:**
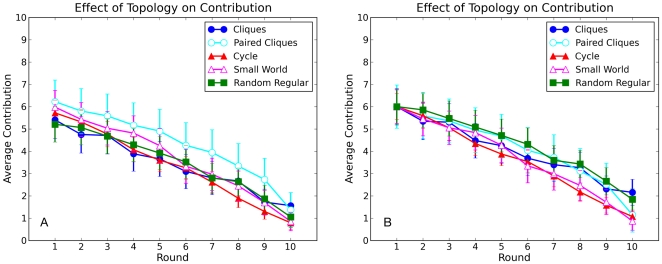
Average contributions per round for each of the five network topologies shown in **Figure 2**. (A) Raw contributions. Error bars indicate 95% confidence intervals.(B) Contribution curves shifted vertically so that they all start at the same point.

In the first set of network experiments all positions in the network were filled by human players recruited from AMT. Because individual contributions tended to vary considerably from one experiment to the next, and different players were likely to play at different times of day, we conducted multiple realizations of the experiment for each topology (see Table 3). The order and timing of experiments was randomly varied between realizations. In total, we conducted 23 experiments over a period of 8 weeks. Figure 4A shows the average contribution for each round, for each of the five topologies. Visually, the average contribution follows a very similar pattern regardless of the network topology. This result is confirmed by a Kruskal-Wallis test [46] on the five distributions (one for each topology) of contributions for each round, which found no significant differences (the smallest P-value is for round 8: H = 6.43, df = 4, P = 0.17). Figure 4A also shows that contribution curves that start higher, relative to other curves, tend to stay above the other curves over the course of the experiments; yet, clearly the first round contributions are random and unrelated to the topology of the network. To see the differences between topologies more directly, therefore, Figure 4B shows the same contribution curves as in 4A, but shifted vertically in order that they have the same initial value. As can be seen, eliminating these initial difference further diminishes the already small differences between topologies.

**Table 3:** The breakdown of realizations per topology is given.

		Paired		Small	Random
	Cliques	Cliques	Cycle	World	Regular
All Human	4	3	8	4	4
Cooperative Seeds, Cover	3	2	4	2	2
Defecting Seeds, Cover	2	2	9	2	2
Cooperative Seeds, Concentrated	N/A	4	5	5	6

The larger number of cycle topology experiments was due to the presence of two outliers: experiments in which uncharacteristically high contributions were registered. The effect of these outliers was to greatly increase the size of the error bars for that topology, thus more realizations were required.

**Figure 5:**
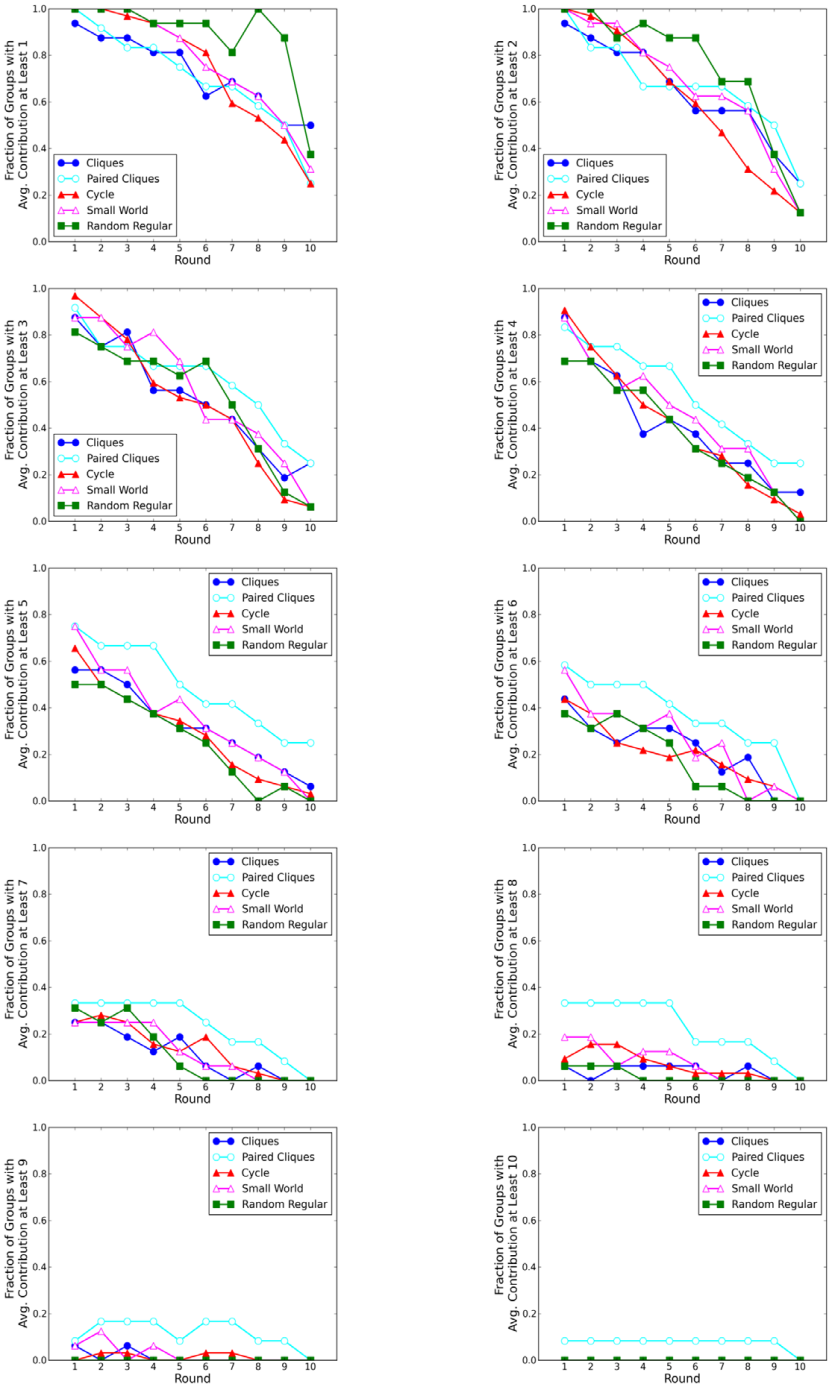
Fraction of groups with average contribution at least 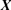, where 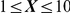.

**Figure 6:**
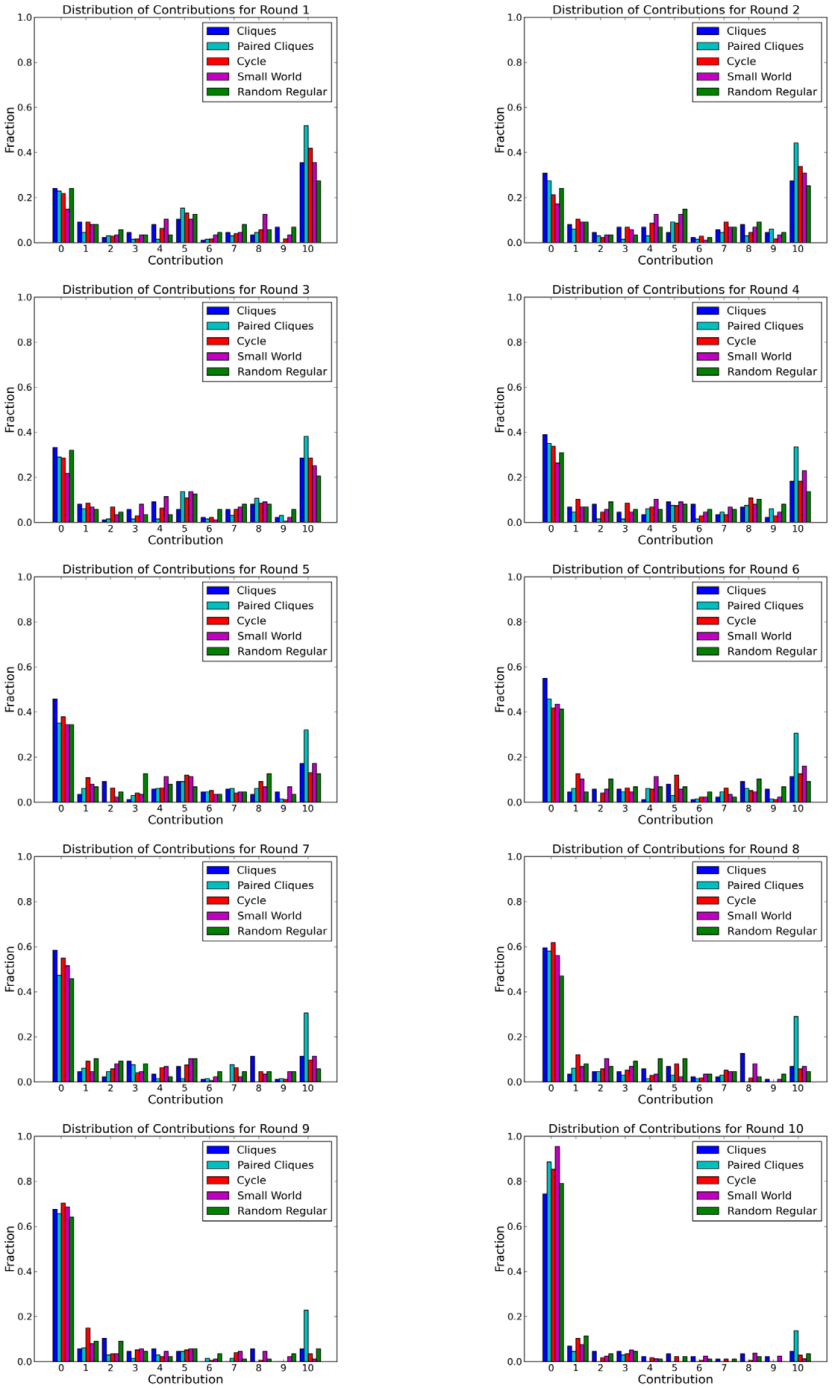
Distributions of individual level contributions across topologies. The distributions vary only slightly as the topology is changed. One realization of the Paired Cliques topology was an outlier; it had a higher then normal number of full contributors.

In addition to considering differences in aggregate contributions, we also checked for differences between topologies both at the level of individual nodes, and for individual “groups” defined as the nodes that are assigned to the same cliques in topology 1 (see colors in Figure 2). As Figure 2 indicates, these groups become progressively less meaningful as the clustering coefficient diminishes: in the Random Regular topology, two nodes in the same group (same color) are no more likely to be connected than nodes of different groups. In spite of these topological differences, however, Figure 5 indicates that they do not impact contributions; specifically, the fraction of groups contributing at least X in a given round is similar for all topologies, and over all rounds. Finally, Figure 6 shows the full distribution of individual level contributions for the five topologies (color coded) over all ten rounds. Although all distributions change dramatically over the course of the game, reflecting the average decline in contributions seen in Figure 4, the changes are similar for all topologies. Thus we conclude that topology does not exert a noticeable impact on contributions at any level: individual, group, or aggregate.

#### Comparison of Results with Cassar (2007)

Cassar [27] conducted a total of 11, 18-player prisoner dilemma experiments on networks of players, where the networks were varied between the following three topologies: a “local” network on which individuals were arranged on a cycle, and each individual was connected to their two nearest and two next-nearest neighbors (i.e. 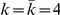 for all nodes); a “small-world” network in which a small fraction of the edges in the cycle were rewired (hence 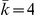, but individual 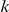 varied); and a “random” network in which individuals were randomly connected (again, 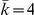, but individual 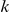 varied). Three realizations of each topology were tested; thus clustering coefficient varied between 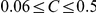, depending on topology, and path length varied between 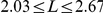, where the local topology had the highest 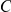 and 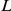, the random topology had the lowest, and the small-world topology was intermediate. Cassar found that cooperation in the small-world topology was significantly lower than either the local or the random topology (Table 5, p. 224 in [27]). She also found that in a logit model, the terms for C and L were negative and positive respectively, and both were significant (Table 10, p. 227 [27]).

At first glance, these findings appear to contradict our own; however, we note that the differences reported as significant in Cassars Table 5 are between cumulative contributions, over the 80 rounds of the experiments. Yet as noted above, and also by Cassar (see her Footnote 13), if the contributions in one realization start at a higher level than other realizations, they tend to stay above the other realizations for the duration of the experiment. This suggests that contributions across consecutive rounds are unlikely to be independent. Combining contributions over many rounds therefore artificially amplifies the differences, leading to the appearance of statistical significance where none may exist. In fact, as Table 5 in [27] itself makes clear, the final (and also average) difference between topologies is roughly the same as the initial difference (period 1–20); thus essentially all of the difference can be explained in term of initial contributions, which are by construction unrelated to the network topology. Second, the significance of the NetworkClustering and NetworkLength coefficients in the PD1 logit model (Table 10 in [27]) is marginal and disappeared when other factors, such as the 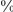 cooperation in the previous experiment (PD2) or dummy variables for the session (PD3) were included. If simply controlling for the session in which a game was conducted eliminates the significance of a coefficient, then it would seem that any claims to significance ought to be regarded with caution. On closer inspection, therefore, Cassar's results are probably consistent with ours—that, is differences in contribution levels between network structures are not significant.

Although Cassar's results on how network structure impacts contribution levels in public goods games may be ambiguous, they do support our claim that the theoretical arguments above [16], [21]–[23] have led researchers to suspect that network structure should matter. Specifically, intuition and simulation results suggest that when conditional cooperators are allowed to interact preferentially, i.e. in networks that exhibit high clustering, they ought to reinforce each other, thereby sustaining higher contributions for longer than in randomly connected networks which have low clustering. Likewise, the contagion argument suggests that clusters of high contributors ought to exert a positive impact on the contributions of neighbors who are not in the cluster, thereby promoting the spread of cooperation. If in fact, network structure does not impact contributions, then one or both of these two arguments must be invalid. To differentiate between these possible explanations, we conducted two additional series of experiments, which we describe in turn.

### Testing for Conditional Cooperation

In the first series, comprising 30 experiments over 4 weeks, we followed the same design as above, but with the key difference that in each experiment four nodes were selected, one from each group (indicated with a filled circle in Figure 2), and their contributions were all artificially fixed either at 10 (the “cooperative” condition) or 0 (the “defection” condition) for all rounds. We emphasize, that these players were played by a computer, not by subsidizing real players, where we did not explicitly disclose to subjects that their neighbors might not be played by other human players. Behavioral scientists of different traditions have varying attitudes with respect deceptive manipulations: experimental economists view them as unacceptable in principle, whereas psychologists practice them when the research benefit outweighs any harm caused to subjects. In our case, subjects were exposed to minimal harm; thus we viewed the benefit of being able to establish clear causal relations as justifying the manipulation. Potentially the issue could have been avoided by including a statement in the instructions to participants to the effect that “from time to time certain positions may be played by automated agents rather than humans.” However, we do not believe that the inclusion of such a statement would have affected the results.

Following the above procedure, we were able test the conditional cooperator hypothesis by directly measuring the positive/negative influence of unconditional cooperators/defectors on their immediate neighbors. We note that with the exception of the random regular network, the seed players were arranged in order to cover the network, meaning that each human player was adjacent to precisely one seed player; in addition, each human player was connected via two-step paths to all four seed players (in the random regular case, a perfect cover arrangement did not exist for the selected network; thus a close approximation was used instead). An advantage of this arrangement, which we call the “cover” condition, is that all human players were subjected to the same experimentally manipulated influence, both direct and indirect.

**Figure 7:**
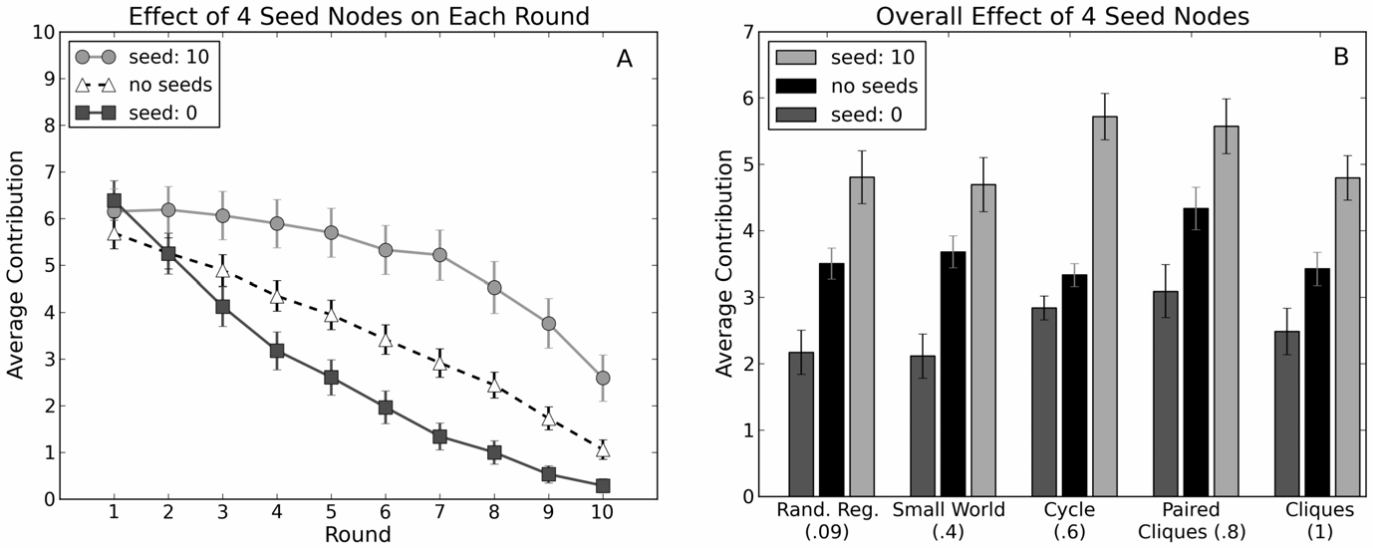
Contributions for cover-seed experiment. (A) Average contribution per round for the cooperating and defecting conditions averaged over all realizations and all topologies. (B) Overall average contribution for each topology under the cooperating, defecting and all human conditions. The clustering coefficient for each network is listed in parenthesis. In both panels error bars indicate 95% confidence intervals.

Figure 7A shows that in all topologies, the presence of cooperating seeds stimulated consistently higher aggregate contributions from the remaining 20 players, while the presence of defecting seeds had the opposite effect. Possessing a high (or low) contributing neighbor therefore did increase (or decrease) the average contribution levels; thus our subjects were indeed behaving as conditional cooperators. Nevertheless, Figure 7B shows that the effect of the seed players was not consistently bigger in the graphs with the highest clustering. For example the effect of the seed nodes in the Cliques network, which had the maximum number of triangles incident on each node, was very similar to the effect of the seeds nodes in the Random Regular network, which had fewer than 1/10th as many triangles. This result implies that two nodes that form a triangle with a cooperating (or defecting) seed do not have an appreciably larger (or smaller) average contribution level then two disconnected nodes with a cooperating (or defecting) seed neighbor in common. Mutual reinforcement of the contributions among the neighbors of a seed node is largely absent, whether or not there is an edge between the neighbors.

**Figure 8:**
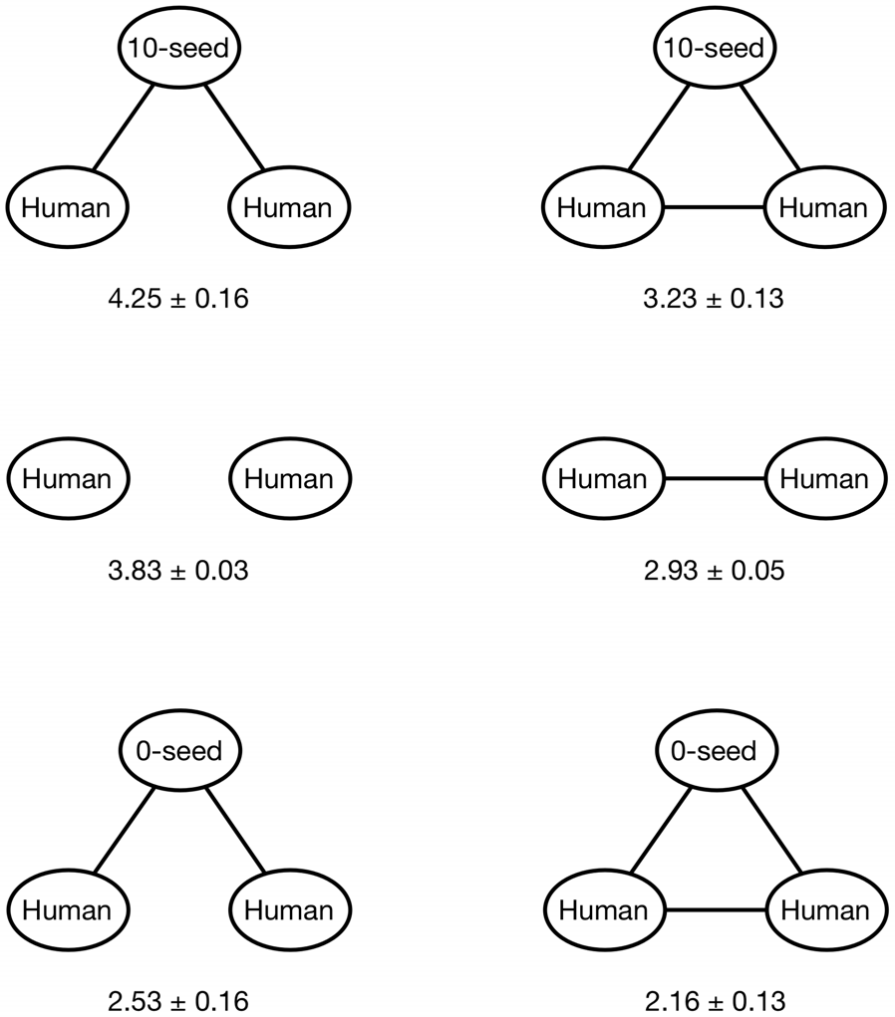
The average pair-wise difference of the human contributions in each of the subgraphs pictured.

Is there in fact any effect of increasing the number of triangles in the network? To answer this question, Figure 8 compares the difference in contributions of pairs of players that (a) are adjacent versus not adjacent, and (b) share a positive or negative seed as a neighbor versus no neighboring seed. Comparing the left column to the right column shows that adding an edge to a disconnected pair of edges increased the similarity between their contribution levels. It also shows that completing a triangle between two human players and a seed node also increased the similarity of the contributions of the humans. Thus, increasing the number of triangles in the network did indeed increase coordination within the neighborhoods of the seeds. We emphasize, however, that the coordinating influence of triangles cuts both ways by increasing contributions in the presence of cooperating neighbors and diminishing them in the presence of defecting neighbors; thus increased coordination among triangles of players does not correspond to increased contribution levels. Put another way, players do cooperate conditionally, but the negative effects of conditional cooperation counteract the positive effects such that the net result is independent of local clustering.

### Testing for Contagion

As noted above, another possible explanation for the lack of impact of network topology on aggregate contributions is the absence of contagion. That is, even if players do behave as conditional cooperators, both with respect to the artificial seeds and also the other neighbors of seeds, possibly these effects are not strong enough to propagate beyond the immediate neighborhood of a cooperation seed. Unfortunately, the above experiment allows us to draw only limited conclusions regarding contagion. Since the cover arrangement of seeds meant that all human players were subjected to the same potential influence, both direct and indirect, we did not experimentally manipulate the level of positive/negative influence at different distances from human players.

**Figure 9:**
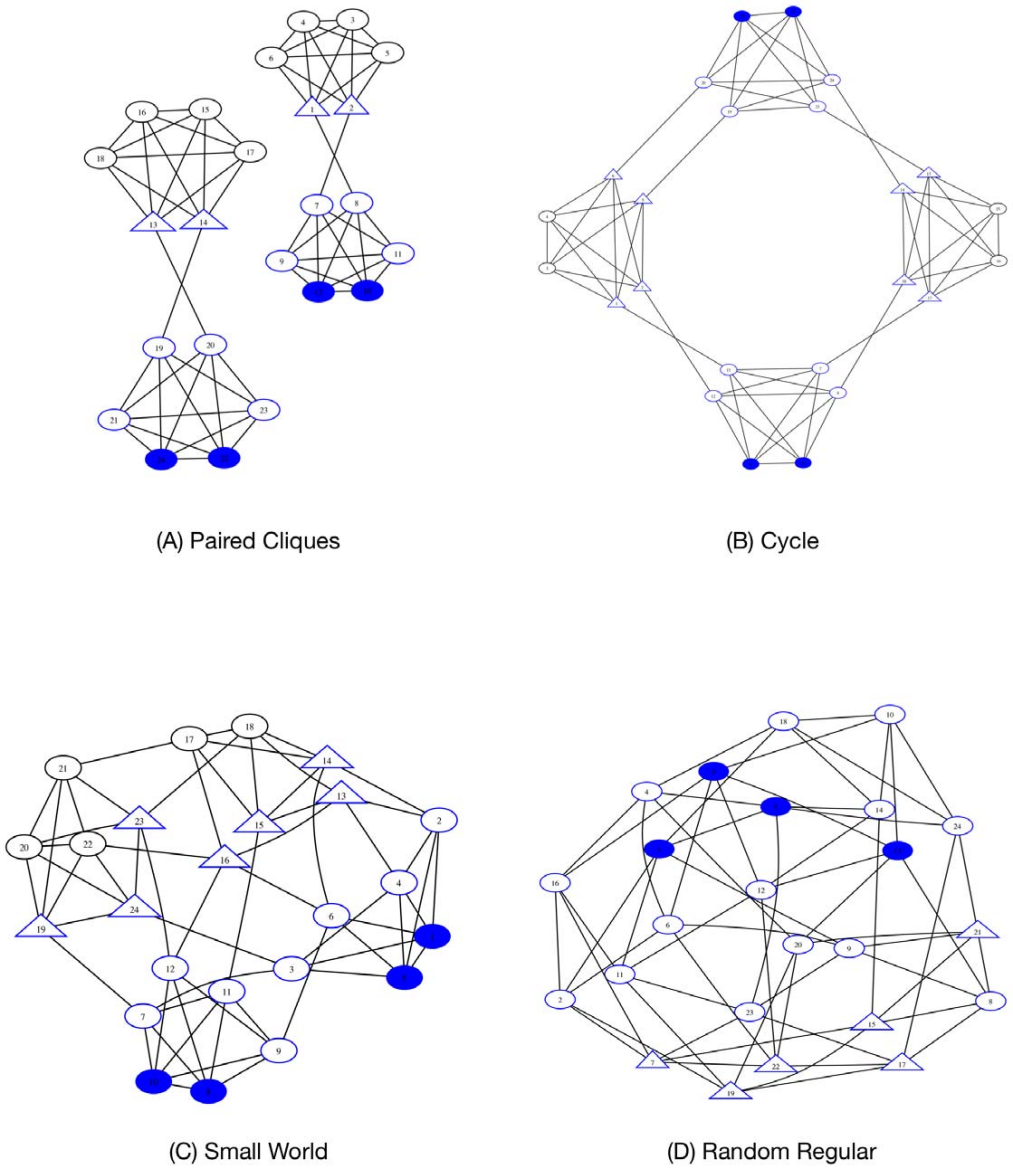
Cooperating seeds in the concentrated seed experiments. The blue, filled-in nodes were used as seed nodes in the concentrated arrangement (see text for details). Oval shaped nodes that are outlined in blue are directly connected to at least one seed node. Triangular nodes are two hops from at least one seed node. In each topology two of the seeds in the concentrated arrangement were also seeds in the cover arrangement.

To further test for the possibility of contagion, therefore, we conducted a third series of 20 experiments over 2 weeks, in which we kept the number of unconditionally cooperating seeds constant at four per network (we did not introduce unconditional defectors in these experiments), but concentrated them together into two adjacent pairs (see Figure 9). This arrangement of seeds, which we call the “concentrated” condition, therefore exposed some human players to two unconditional cooperators as immediate neighbors, while others were not exposed to any seeds directly, but were connected indirectly to the seeds via a human intermediary. Since the Cliques topology did not allow for this type of arrangement we excluded it from these experiments. If positive contagion were present in the network, we would expect to see nodes at distance two from the seeds increase their contributions relative to the all-human (i.e. no seeds) condition. Moreover, the premise of conditional cooperation would also lead us to expect that immediate neighbors would increase their contributions relative to the cover-seed condition.

**Figure 10:**
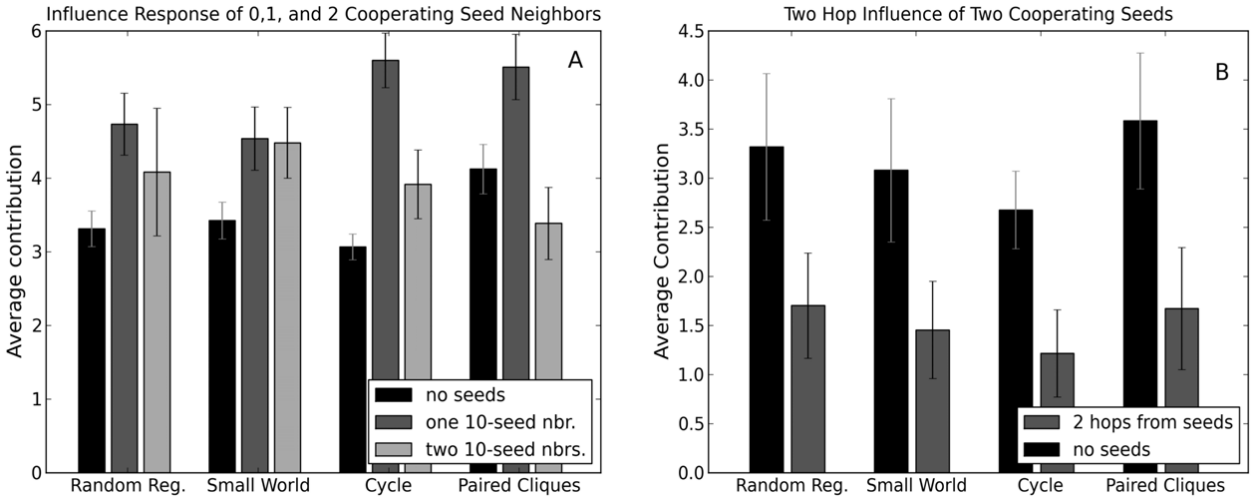
Contributions for concentrated-seed experiment. (A) The average contribution of human players neighboring 0, 1 or 2 cooperating seed nodes. (B) The average contribution of the human players 2 hops from 2 seed nodes compared to the average contribution of the corresponding nodes in the all human experiments. In both panels error bars indicate 95% confidence intervals.

Surprisingly, our results contradicted both these expectations. First we found that nodes who were directly connected to two cooperating seed nodes contributed more than players who were not attached to any seed nodes, but less than players who were attached to only one seed node (both computed from previous experiments) as shown in Figure 10A. These results suggest that although many players do respond positively to the introduction of unconditional cooperators, the presence of too many unconditional cooperators invites free riding. Conditional cooperation, that is, appears to be subject to at least two distinct conditions that are in tension with one another: on the one hand, individuals do not want to contribute unless others are contributing; but on the other hand, if others contribute too much, the temptation to free ride overrides their inclination to reciprocate. In spite of this result, it is nevertheless the case that immediate neighbors of cooperating seeds did on average contribute more than in the no-seed condition. Assuming that the remaining players (i.e. at distance two from the seeds) also cooperate conditionally, one would expect that the increased contributions associated with the neighbors of a fully contributing seed would generate contagious effects leading to increased contributions among these nodes as well. Yet these effects were not apparent. Quite to the contrary, in fact, Figure 10B shows that the two-step neighbors of the cooperating seeds contributed slightly less than the nodes in the corresponding network positions contributed in the all-human experiments.

#### Testing for Learning Effects

**Figure 11:**
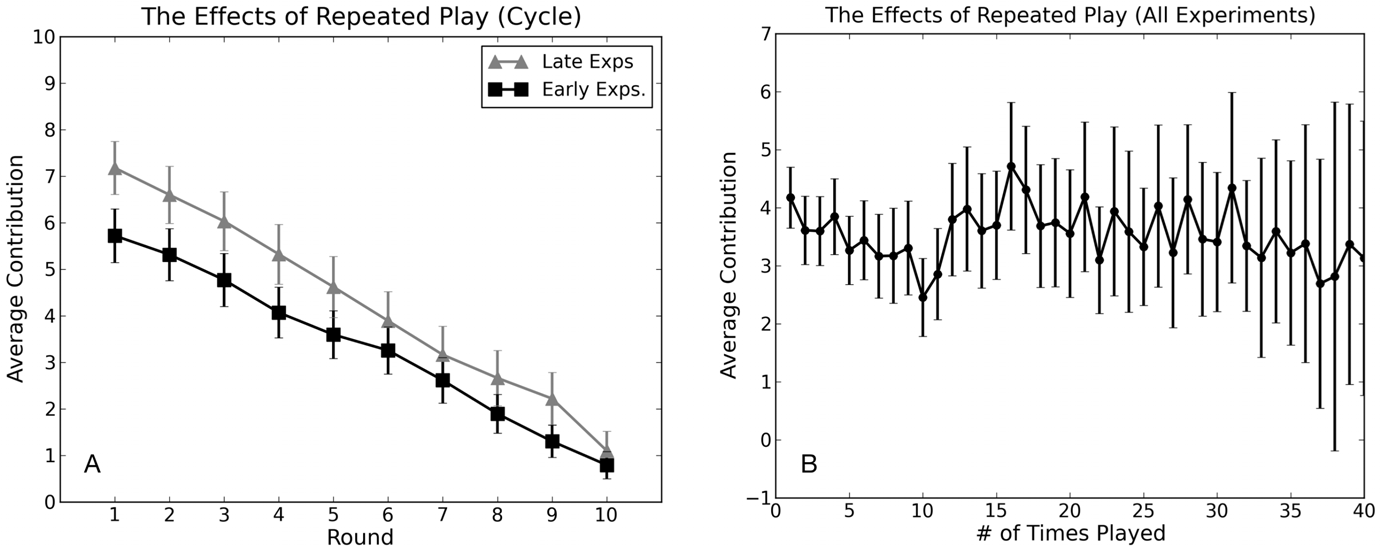
Checking for the effects of learning. (A) Comparison on the Cycle topology with all human players between experiments conducted early in our study and at the end of our study. (B) Average contribution levels as a function of how many times an individual has played.

To check that this unexpected reduction in contributions did not reflect a systematic overall shift from higher to lower contributions over the course of dozens of experiments involving our panel, we ran an additional series of all-human experiments, finding that average contributions had, if anything, increased slightly relative to the earlier round of all-human experiments (see Figure 11A). We also studied average contributions as a function of the number of games played by individual subjects, finding that experienced players who have played as many as 40 games did not contribute on average, more or less than inexperienced players (see Figure 11B). Moreover, we tested for selection effects by comparing the complete history of average contribution levels of those who chose to play many times to the overall subject pool and did not find a significant difference. Thus we conclude that the reduced contributions observed in the concentrated seed experiments are not explainable either in terms of a systemic over-time shift in player behavior, the presence of experienced players contributing less, or a higher return rate of more cooperative players. We also note that although experienced players have been used in previous experiments [31], it is unusual to allow subjects to play upwards of 30 times over a period of months. Previously it has been unclear whether or not such players would learn over time to play differently, thereby systematically biasing the results. Figure 11 is therefore reassuring in that it shows no evidence of such a systematic bias.

#### Comparison of Results with Fowler and Christakis (2010)

Finally, we note that our finding that positive contagion does not occur in public goods games on networks appears to contradict a recent claim by Fowler and Christakis [20] mentioned earlier. The authors claim that cooperative cascades take place on networks of individuals playing a linear public goods game, and that evidence of contagion persists for up to three steps, leading them to hypothesize a “three degrees of influence” rule. We note, however, an important difference between the networks studied by Fowler and Christakis and those that we have studied here. Specifically, Fowler and Christakis reanalyzed data from Fehr and Gachter [32] (the same results that we replicated in our preliminary experiments described above) in which groups of 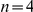 players were randomly reassigned to new groups after each round. Whereas in our networks, all individuals appear just once and play with same set of neighbors each turn, in [20] each individual appears 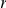 times (where 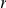 is the number of rounds of the experiment) and plays with a different set of neighbors each time. As a result, the measure of network distance in [20] does not map precisely to the conventional meaning of network distance, which is the meaning that we have adopted here, but rather refers at least in part to the relation between an individual's present and past states. Although this unconventional definition of distance makes the two sets of results difficult to compare, the main finding in [20], that individuals who belonged to higher contributing groups in round 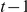 contributed, on average, more in round 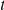, seems consistent with our observation that initially high contributions tend to persist over time. We also note, however, that it was precisely to separate the effects of persistence from “true” contagion, in the sense that an effect due to a single individual propagates to a remote individual along a series of network ties, that we designed the concentrated seed experiment. And as the results from that experiment make clear, neither persistence nor even conditional cooperation (as demonstrated in the cover seed design) are sufficient to generate contagion in this sense. Given these results, we conclude that although the effects of higher neighbor contributions may well persist for up to three rounds, the most intuitive interpretation of the “three degrees of influence” rule—namely that higher contributions spread from individual to individual in a static network for up to three steps—is not supported.

## Discussion

Returning to our original motivation, theoretical arguments in favor of an association between network structure and cooperation invoke two related ideas: first, that individuals are conditional cooperators, increasing their contributions in response to the increased contributions of their neighbors; and second, that positive effects of conditional cooperation should propagate through the network via a process of contagion. In this paper, we have tested the effects of network topology on contribution levels in a standard public goods game, finding no significant effects. In addition, we conducted two separate rounds of experiments—one to test for the presence of conditional cooperation, and the other to test for the possibility of positive contagion. Although we do find strong evidence of conditional cooperation, we do not find evidence of positive contagion in the standard sense of multi-step propagation along a sequence of ties in a static network.

Our explanation for these results is that the theoretical arguments cited above emphasize the positive aspect of conditional cooperation, yet conditional cooperation implies not only that players increase their contributions in response to cooperative neighbors, but also that they decrease their contributions in response to defecting neighbors. Although it is the case that highly clustered networks offer more opportunities for positive effects to reinforce each other than random networks, they also offer more opportunities for negative effects to reinforce each other as well. By contrast, in random graphs where there is very little clustering, neither cooperation nor defection get reinforced and seeds act as influence blockers preventing either positive or negative influence from propagating among neighbors.

As stated in the introduction, in the case of coordination games, if node A chooses an action that results in a lack of coordination with neighbor B, then B has a clear incentive to change its action. In turn, if this results in a lack of coordination with C which is a neighbor of B and not A, this can result in contagion. In a cooperation setting, B need not change its action in response to A because the incentives do not enforce such a tight coupling of neighbors actions. This leads to an interesting open question—under what theoretical conditions should one expect to see contagion over networks with fixed neighbors? In demonstrating that not all dynamic games on networks exhibit contagion we hope that our findings will provoke new theoretical hypotheses along these lines, as well as new experiments to test them.

Moreover, even in the absence of contagion, our observations also show how an outside entity might stimulate cooperation in a network by subsidizing targeted individuals to cooperate or by inserting unconditionally cooperative players into the network. We emphasize that unlike other known strategies for stimulating cooperation, such as allowing punishment [5] or reward [8], or introducing sanctioning institutions [7], this mechanism does not change the game by giving players another action, but instead exploits the network on which the game is being played. As Table 1 shows, in the cover experiments the positive intervention was cost-effective in four out the five topologies. More specifically, the expected cost of subsidizing players, i.e. the additional contributions of the four seeds over their average contribution in the no-intervention case, was less than the total marginal increase in contributions from the remaining twenty individuals. These results therefore provide empirical support for earlier theoretical work [47] which proposed that seeding or inducing cooperation among focal actors may generate positive effects on the network. Our work also suggests where to place the seed nodes for maximum effect. The absence of positive contagion—along with the negative marginal effect on neighbors of multiple unconditionally cooperating seeds—implies that the impact of cooperative seeds is maximized by spreading them widely across many groups, thereby maximizing the total number of human players exposed directly to seeds.

In concluding, we note that in addition to their substantive relevance, the experiments discussed here also demonstrate the possibility of web-based behavioral experiments involving the simultaneous presence of many players (see also [44]). Although experiments of this nature and scale have been conducted in physical labs [24]–[27], web-based “virtual labs” exhibit two important advantages over their physical counterparts: first, experiments can be run faster and more efficiently (e.g. we ran 113 experiments costing roughly $1.50 per subject per experiment); and second, although our panel size restricted the current study to networks of 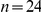, in principle this limit can be raised arbitrarily, allowing for the study of much larger networked systems. The speed, efficiency, and scalability of web-based experimentation should allow researchers to extend the current study in numerous directions: how would contributions be affected by giving players more information about the network, or providing players with feedback, or allowing players to rewire their network ties? And how do all these effects scale with the size and density of the network? In addressing these questions, and others, we anticipate that web-based platforms like that provided by AMT will become an increasingly valuable tool for understanding the dynamics of human cooperation, and for experimental social science in general.

## Materials and Methods

This section provides additional details on the Investment Game experiment, conducted on Amazon's Mechanical Turk (AMT). All participants were recruited on AMT by posting a HIT for the experiment, entitled “The Investment Game”, a neutral title that was accurate without disclosing the purpose of the experiment. Before launching the experiment, we submitted to and complied with Yahoo!'s internal human subjects review process. A letter certifying the approval of our experiment has been filed with PLoS One. All data collected in the experiment could be associated only with participants' Amazon Turker ID, not with any personally-identifiable information; thus all players remained anonymous.

### Ethics Statement

Before participating, all subjects were required to read and acknowledged the following terms of use agreement (equivalent to an Informed Consent Statement).

#### The Investment Game Terms of Use

You will be paid $0.50 as a base rate plus more depending on your ability to play the game If you have any questions at any time, please contact: Siddharth Suri at Yahoo! Research, 111 W. 40th St., New York, NY, or by email at suri@yahoo-inc.com By clicking the “I Agree” button below you affirm that you have read and understood the following Yahoo! Research Investment Game Terms of Use and Investment Game description and agree to comply with and be bound by its terms. YAHOO! RESEARCH INVESTMENT GAME TERMS AND CONDITIONS

1. Welcome to the Yahoo! Research Investment Game (“Project”). This Project is a game of skill, not a game of chance. By participating in the Project you are entering into a legally binding agreement with Yahoo!, Inc., (“Yahoo!, “we,” “our,” and “us”). This agreement is comprised solely of these Terms and Conditions (“Agreement” or “Terms”), including anything explicitly incorporated by reference. If you do not agree to these Terms, please do not participate.
2. The Project is offered to individuals registered as “workers” with Amazon, Inc.'s “Mechanical Turk” service (http://www.mturk.com/mturk/welcome).
3. Your participation in the Project as a worker is governed by Amazon, Inc.'s Mechanical Turk's conditions of use (http://www.mturk.com/mturk/conditionsofuse) in addition to the following Yahoo! terms:
4.
  - Description of Project. The Yahoo! Research Investment Game is intended to collect data on how well people play this game.
  - Work Product/Ownership. You agree to perform the tasks provided in the Project and to be compensated for the completion of each task as set forth in C below. You also agree that Yahoo!, and not You, shall own all work product from your participation in the Project.
  - Payment. You will be paid $0.50 plus a bonus depending on your skill level for the game. All payments will be made to You through the Mechanical Turk service as detailed in the Mechanical Turk conditions of use.
  - Relationship of the Parties. The Parties are independent contractors. Nothing in these Terms shall be construed as creating any agency, partnership, or other form of joint enterprise between the Parties and neither Party may create any obligations or responsibilities on behalf of the other Party.
  - Termination.
    - By You. You may terminate Your participation in the Project by clicking the Return HIT button at any time.
    - By Yahoo!. We may suspend or terminate the Project at any time, with or without notice, for any reason or no reason. In the event of such termination, Yahoo! will pay You for all tasks fully completed by You prior to termination.
  - Contact. If you have any questions at any time, please contact Siddharth Suri at Yahoo! Research, 111 W. 40th St., New York, NY, or by email at suri@yahoo-inc.com
  - Confidentiality. You will not disclose or use Yahoo!'s Confidential Information. “Confidential Information” means any information disclosed or made available to You by Yahoo!, directly or indirectly, whether in writing, orally or visually, other than information that: (a) is or becomes publicly known and generally available other than through Your action or inaction or (b) was already in Your possession (as documented by written records) without confidentiality restrictions before you received it from Yahoo!. Confidential Information includes, but is not limited to, all information contained within the Project, these Terms, the Policies, and any other technical or programming information Yahoo! discloses or makes available to you.
  - Indemnity. You will defend, indemnify and hold harmless Yahoo! Inc., and its affiliated companies, (“Indemnified Parties”) from and against any and all claims, liabilities, losses, costs, and expenses, including reasonable attorneys' fees, which the Indemnified Parties suffer as a result of claims that arise from or relate to your activities under or in connection with this Agreement, including but not limited to claims that allege or arise from: (i) a violation a third party's right of privacy, or infringement of a third party's copyright, patent, trade secret, trademark, or other intellectual property rights, (ii) any breach of your obligations, covenants, warranties or representations as set forth in this Agreement, including any breach of any applicable policies, (iii) any violation of applicable laws, rules, and regulations by you, including, without limitation, privacy laws, and (iv) any breach of this Agreement. You shall not enter into any settlement that affects any Indemnified Party's rights or interest, admit to any fault or liability on behalf of any Indemnified Party, or incur any financial obligation on behalf of any Indemnified Party without that Indemnified Party's prior written approval.
  - No Warranty. YOU EXPRESSLY AGREE TO THE FOLLOWING WARRANTY DISCLAIMER. YOU ARE PARTICIPATING IN THE PROJECT AT YOUR OWN RISK. YOU REPRESENT AND WARRANT THAT BY PARTICIPATING IN THIS PROJECT THAT YOU WILL COMPLY WITH ALL APPLICABLE LAWS. THE PROJECT AND EVERYTHING PROVIDED UNDER THIS AGREEMENT IS PROVIDED “AS IS.” YAHOO! DOES NOT WARRANT THAT THE PROJECT WILL OPERATE UNINTERRUPTED OR ERROR-FREE. YAHOO AND ITS LICENSORS ARE NOT RESPONSIBLE FOR ANY CONTENT PROVIDED HEREUNDER. TO THE EXTENT ALLOWED BY LAW, YAHOO! AND ITS LICENSORS MAKE NO WARRANTY OF ANY KIND, WHETHER EXPRESS, IMPLIED, STATUTORY OR OTHERWISE, INCLUDING WITHOUT LIMITATION WARRANTIES OF MERCHANTABILITY, FITNESS FOR A PARTICULAR PURPOSE, AND NONINFRINGEMENT. YAHOO! MAKES NO WARRANTY AND NO REPRESENTATION ABOUT THE AMOUNT OF MONEY YOU WILL EARN THROUGH THE PROGRAM. THIS WARRANTY DISCLAIMER SHALL APPLY TO THE MAXIMUM EXTENT PERMITTED BY LAW.
  - Limitation of Liability. YOU EXPRESSLY AGREE TO THE FOLLOWING LIMITATION OF LIABILITY. YAHOO! WILL NOT BE LIABLE FOR ANY LOST PROFITS, COSTS OF PROCUREMENT OF SUBSTITUTE GOODS OR SERVICES, OR FOR ANY OTHER INDIRECT, SPECIAL, INCIDENTAL, EXEMPLARY, PUNITIVE OR CONSEQUENTIAL DAMAGES ARISING OUT OF OR IN CONNECTION WITH THIS AGREEMENT, HOWEVER CAUSED, AND UNDER WHATEVER CAUSE OF ACTION OR THEORY OF LIABILITY BROUGHT, EVEN IF YAHOO! HAS BEEN ADVISED OF THE POSSIBILITY OF SUCH DAMAGES. YAHOO! WILL NOT BE LIABLE FOR DIRECT DAMAGES IN EXCESS OF ANY AMOUNT THAT YAHOO! HAS ALREADY PAID TO YOU FOR YOUR PARTICIPATION IN THE PROJECT. IF YOU ARE DISSATISFIED WITH ANY ASPECT OF THE PROJECT, OR WITH ANY OF THESE TERMS OF USE, YOUR SOLE AND EXCLUSIVE REMEDY IS TO DISCONTINUE YOUR PARTICIPATION IN THE PROJECT. This limitation of liability shall apply to the maximum extent permitted by law.
  - No Public Statements. You may not issue any press release or other public statement regarding the Agreement, Yahoo!, and/or Yahoo! Inc.s affiliates, or partners or advertisers without the prior written consent of an authorized person at Yahoo!.

I AGREE

### Participant Instructions

After accepting the HIT and agreeing to the terms of use, participants were provided with the following instructions (adapted from [32]).

#### Welcome to the Investment Game!

Because the amount of money you can earn depends on your decisions in the game, it is important that you read these instructions with care. At the end of the instructions there is a quiz to ensure that you understand the instructions. You will not be paid for the HIT unless you correctly answer these questions.

#### Overview

In the Investment Game you will be placed in a network with 23 other Turkers; however, you will only “see” a subset of the total network-those players to whom you are connected directly. These players will be called your “neighbors”. Both the total network and your neighbors will remain fixed throughout the game.

Once the network is populated with Turkers, the game will proceed over the course of 10 “rounds”. During each round you and your neighbors (i.e. the Turkers directly connected to you in the network) will choose how much to contribute to an abstract project. Then this project generates a “payoff” that will then be split equally among you and those who are directly connected to you. Your total payoff for the game is the sum of your payoffs from each round.

During the game we will not report your earnings in terms of dollars and cents but rather in terms of points. At the end of the game the total amount of points you have earned will be converted to dollars at the rate of 1 point  =  2 cents. The amount you earn from the game will be the bonus for this HIT. You will earn the base rate of 50 cents for this HIT by correctly answering the quiz at the end of these instructions.

#### How the game works

1. In each round we give you an “endowment” of 10 points.
2. You decide how many points you want to contribute to the project by typing a number between 0 and 10 in the input field and then clicking the submit button. Please note that by deciding how many points to contribute to the project, you also decide how many points you keep for your self, this is (10 - your contribution) points. Also note that once you have submitted your contribution you cannot go back and change it.
3. In the first two rounds you have 45 seconds to make you contribution. In the remaining rounds you have 30 seconds. If you do not make a contribution before the end of a round, the system will make one for you and you will not earn any points for that round.
4. Your income from each round consists of two parts:
  - the points which you have kept for yourself (“income from points kept”).
  - “income from the project”, which is 0.4 x the total contribution that you and your neighbors made to the project.Your income in points from a round is therefore: Income from points kept + Income from the project  =  (10 - your contribution to the project) + 0.4*(total contributions you and your neighbors made to the project)
5. The income of each person in the network (including your neighbors) is calculated in the same way.

#### Four Examples of Payoffs

1. Suppose you have four (4) neighbors, and each of you contributes the maximum allowable of 10 points. The sum of the contributions you and your neighbors (those who are directly connected to you) is 50 points, and so each member of the group receives an income from the project of: 0.4*50 = 20 points. Meanwhile your income from points kept  =  0 (because you did not keep any), and so your total income  =  0+20 = 20 points.
2. Alternatively, suppose that each player contributes two (2) points. Then the total contribution to the project is 10 points, and each member of the group receives an income from the project of: 0.4*10 = 4 points. Because you contributed two of these points then your income from points kept is eight (8), and your total income  =  8+4 = 12 points.
3. Next, say you contribute two (2) points and all your neighbors contribute ten (10) points, the total contribution is 42 points, and the income that each player receives from the project is 0.4*42 = 16.8 points. Because you contributed two (2) points, your kept income is eight (8), and your total income  =  8+16.8 = 24.8 points.
4. Finally, say you contribute ten (10) points, and all your neighbors contribute two (2) points, the total contribution is 18 points, and the income that each player receives from the project is 0.4*18 = 7.2 points. Because you contributed ten (10) points, your kept income is zero (0) points and your total income  =  0+7.2 = 7.2 points.

#### Important Points to Note

1. For each point that you decide to keep for yourself, your income for that round will increase by one point.
2. For each point you contribute to the project, the total contribution to the project will rise by one point, and your income from the project will rise by 0.4*1 = 0.4 points.
3. For each point you contribute to the project, the income of your neighbors will rise by 0.4 points each. For example, if you have 4 neighbors then a one point contribution by you will raise the total income of you and your neighbors by 5*0.4 = 2.0 points.
4. Finally, say you contribute ten (10) points, and all your neighbors contribute two (2) points, the total contribution is 18 points, and the income that each player receives from the project is 0.4*18=7.2 points. Because you contributed ten (10) points, your kept income is zero (0) points and your total income = 0+7.2=7.2 points.

### Participant Quiz

Finally, participants were required to pass a quiz, thus demonstrating that they had understood the instructions.

#### Quiz

To make sure you have read and understood the instructions, you must answer the following questions correctly. If you answer any questions incorrectly, you will get a second chance. If you answer a question incorrectly twice, you will not be allowed to play the game and will not receive payment for the HIT. The answers to all of the questions below are in terms of points. Please accept the HIT before beginning to fill out the form.

In questions 1–4, assume you have 5 neighbors and you and your neighbors have an endowment of 10 points each.

1. If nobody (including yourself) contributes any points to the project what would your total income be?
2. If everyone (including yourself) contributes all 10 points to the project, would your total income be?
3. Say together your neighbors contribute a total of 25 points to the project.
  - If you do not contribute any points to the project what would your total income be?
  - If you contribute an additional 5 points to the project what would your total income be?
4. Say you contribute 8 points to the project.
  - What would be your income if your neighbors contributed a total of 12 points to the project?
  - What would be your income if your neighbors contributed a total of 32 points to the project?
